# Health care without medicine: the impact of war on Sudan’s pharmaceutical manufacturing and supply

**DOI:** 10.1186/s13031-025-00667-z

**Published:** 2025-05-09

**Authors:** Aly Verjee, Thouiba Hashim Galad, Ruoh-Yeng Chang

**Affiliations:** 1 University of Gothenburg, Gothenburg, Sweden; 2 Doha, Qatar; 3 University of British Columbia, Vancouver, Canada; 4 University of Alberta, Edmonton, Canada

**Keywords:** Sudan, Conflict, Pharmaceutical industry, Essential medicines

## Abstract

**Background:**

Armed conflict in Sudan since April 2023 has led to the widespread disruption of health care services, the destruction of public and private health care infrastructure nationwide, and the targeting of health care personnel. This study examines the impact of the conflict on a less publicized sector of Sudan’s health system: the national pharmaceutical manufacturing and supply chain.

**Methods:**

Research interviews were conducted with representatives of 4 companies engaged in pharmaceutical manufacturing and/or supply in Sudan, in addition to a review of primary information from medical regulators and industry associations. Based on these interviews, 3 case studies are presented; insights from the fourth interview inform the study overall.

**Results:**

All 4 companies reported significant impacts on their operations, as well as on other companies in the pharmaceutical sector. Domestic pharmaceutical manufacturing has effectively come to a halt, leading to the non-availability of certain drugs in parts of the country. Manufacturers have shifted course to focus on imports, but heavy losses of domestically stored inventory and an inability to distribute medicines to large parts of the country have had a significant impact on pharmaceutical supply to retail pharmacies, public and private sector health facilities, and the National Medical Supplies Fund (NMSF).

**Conclusions:**

Sudan’s pharmaceutical manufacturing industry has been devasted. Although less visible than hospitals and medical personnel, the effects on the pharmaceutical sector are consequential for Sudan’s health sector, particularly for chronic conditions.

## Introduction

In April 2023, a brutal war began between the Sudanese military and the Rapid Support Forces (RSF), a militia formerly aligned with government forces. The war was exacerbated by decades of militarization, racism, and inequality in Sudan, a country of nearly 50 million people. Sudan is sadly accustomed to violent conflict, having spent more years in war than peace since becoming an independent state in 1956 [1]. However, the current war differs from earlier conflicts primarily waged on the country’s peripheries: the present conflict has affected all regions of the country, including Sudan’s most populous, richest, riverine heart, comprising the capital area of Khartoum [2].

Sudan’s deeply unequal society, with wealth, power, and infrastructure heavily concentrated in Khartoum, makes the capital region especially critical in the health care sector. Most specialized health facilities and medical training opportunities are located in Khartoum, as is a small but significant health supplies and pharmaceutical manufacturing and distribution industry. While attacks on health care workers and health facilities have rightly received attention [3–5], all elements of the country’s health infrastructure have been affected by the conflict, including the pharmaceutical sector [6, 7].

This article examines the war’s impact on Sudan’s pharmaceutical manufacturing, import, and supply. While Sudan relies heavily on imported drugs, local pharmaceutical manufacturing supplies about 30% of the overall needs of the Sudanese health sector, largely focused on producing low-cost essential medicines [8, 9]. About 27 pharmaceutical companies manufacture drugs in Sudan, all of which were based in the Khartoum / Khartoum North area of the capital region [9]. As of 2023, Sudan’s National Medicines and Poison Board (NMPB) licensed 153 different companies to manufacture, import, and/or supply pharmaceutical products in Sudan [10]. Sudan’s pharmaceutical supply chain is truly global, with products licensed for import from at least 30 countries on 4 continents, including Austria, Belgium, China, Egypt, France, Germany, Greece, India, Indonesia, Ireland, Italy, Japan, Jordan, Malaysia, Oman, Pakistan, Philippines, Portugal, Qatar, Saudi Arabia, Slovenia, Spain, Sweden, Syria, Thailand, Tunisia, Turkey, the United Arab Emirates, the United Kingdom, and the United States [10]. In addition, the National Medical Supplies Fund (NMSF) procures drugs for state-owned health care facilities, as well as essential medicines to treat haemophilia, cancer, and kidney disease [11].

Despite the significance of international imports, Sudan’s small but important domestic pharmaceutical manufacturing sector produces many medications for both acute and chronic use, such as antibiotics (e.g. amoxicillin, azithromycin, metronidazole), benzodiazepines (e.g. diazepam), common analgesics (e.g. paracetamol / acetaminophen), nonsteroidal anti-inflammatory drugs (NSAIDs, e.g. ibuprofen and mefenamic acid), cardiac medications (e.g. clopidogrel bisulfate), treatments for diabetes (e.g. vildagliptin), and gastrointestinal upset (e.g. domperidone, loperamide), among others [10, 12]. While this product list is not exhaustive, it demonstrates the diversity of Sudanese pharmaceutical manufacturing across different drug classes with different therapeutic values.

## Materials and methods

### Study design

Given the considerable difficulties of safely and ethically conducting research during armed conflict, which include the displacement and/or inaccessibility of representatives of all companies in the sector, and the need to protect study participants as individuals, this study uses a pseudonymized case study approach.

Interviews with companies variously engaged in the manufacture, import, and distribution of pharmaceutical products in Sudan were conducted in March, April, and May 2024. Neither respondents nor their companies are identified by name, given the ongoing conflict and sensitivities relating to their attempts to continue operations and engage with the host country authorities. Accordingly, all company names have been pseudonymized in this article, and any potentially identifying details, such as exact company locations and production statistics, made non-specific to preclude identification.

### Data collection

Qualitative data was collected from interview respondents in semi-structured interviews conducted in Arabic, with responses then translated into English. Interviews focused on the background to the company’s establishment, its historic focus of operations, before moving to discuss the impact of the current armed conflict on the company’s operations, resources, and infrastructure. Finally, interviews touched on company plans to continue or resume operations.

### Analysis

Interview data was summarized for qualitative analysis, focusing on the experiences of the companies involved and the common trends of cases. Three case studies are presented based on the corresponding interviews; insights from the fourth interview inform the study overall. In this study, no statistical analysis was conducted.

## Results

### Case study 1: the impact of war on pharmaceutical manufacturing: Company A [13]

Company A is a Sudanese company engaged in pharmaceutical manufacturing. Its parent company is based in the Middle East, and is active in the agricultural and industrial sectors, in addition to pharmaceutical products. Company A’s main production site in Sudan is a large facility of more than 6,000m^2^ in the industrial zone of the city of Khartoum North, where some of the heaviest fighting of the conflict took place between the Sudan Armed Forces and the RSF. The factory employed more than 300 staff, and could produce medicines in various dosage forms, including tablets, capsules, oral solids, and liquid suspensions. The factory’s annual production capacity was more than 200 million base unit measurements, and its 2023 production target was 16 million units.

Among the products manufactured in Sudan were two generic antibiotics: a macrolide and a cephalosporin. These antibiotics are commonly used to treat mild to moderate bacterial infections of the skin as well as upper and lower respiratory infections such as pneumonia, as well as genitourinary systems infections. Prior to the war, the factory produced over one million units of these medicines monthly. In total, Company A produced more than 100 items at its Sudanese plant.

With the onset of conflict, the company lost access to the factory site. Its personnel have been unable to enter the facility since the war began. Consequently, manufacturing has stopped completely. Ancillary equipment, like vehicles and computers, were stolen. As Company A’s representative put it, “the war had a devastating impact on our factory operations” [13]. While Company A was not able to specifically quantify its financial losses, the figure appears to run into the millions of dollars. Apart from ongoing production, the war stopped ambitious expansion plans, as well as intentions to export drugs to neighbouring countries like South Sudan that lack domestic pharmaceutical manufacturing entirely.

Even if access to the factory site had been regained, the prevailing security situation would have made it impossible to import raw materials for production from abroad. Consequently, within two months of the war, Company A shifted to importing drugs from Saudi Arabia, by sea, and from Egypt, overland. Import volumes have not fully substituted for what was being produced within Sudan, however.

The Company has also started contract manufacturing in a foreign country where it did not previously have operations, with the intent of replacing the manufacture of medicines previously produced in Sudan. Some of this contract manufacturing is now being transported to Port Sudan on Sudan’s Red Sea coast, for onward distribution.

Historically, Company A directly distributed its products to pharmacies in Khartoum State, while distribution in other areas of Sudan was carried out through agents. Given the ongoing disruption, Company A estimates that its product distribution has fallen to 20% of the volume of its previously planned target for 2023. It has relocated distribution operations to Gezira State with about 40 staff, distributing products solely to the eastern and northern states of Sudan (leaving the central and western states unsupplied).

### Case study 2: the impact of war on pharmaceutical manufacturing: Company B [14]

Company B was established in Sudan approximately 50 years ago, initially through supplying dental products and ancillary goods. About 20 years ago, the company established a plant in Khartoum North, which was largely producing pediatric medicines, including a mucolytic drug used in the treatment of respiratory disorders, cough syrups, and NSAIDs, sometimes in collaboration with another Sudanese pharmaceutical manufacturer. Company B also produced medical equipment, like syringes.

Company B also acted as an agent for prominent European pharmaceutical companies, supplying, among other drugs, medication for kidney failure.

Company B’s factory was hit by heavy fire (it is unclear whether this was from ground-based artillery fire or from an air strike; both were common tactics used during the fighting in the city), leading to the factory’s destruction. Goods and materials held at the factory were either stolen or destroyed. Company representatives are unsure of the status of raw materials used in manufacture, as well as of packaging materials that were stored in the vicinity of the factory. Moreover, company representatives expressed concern at the possible environmental impact, including water contamination, that might have resulted from the attack on the factory. As a representative for Company B noted, “we need to test the water to determine the extent of the [environmental] damage caused by the factory’s presence in the centre of Khartoum” [14].

In part because approximately 30 vehicles belonging to the company were stolen, all distribution of medicine from company stores has stopped. The company’s management has since left the country. Efforts to begin imports from neighbouring countries were unsuccessful.

Like Company A, Company B was not able to specifically quantify its financial losses, but again the impact appears to be significant.

### Case study 3: the impact of war on pharmaceutical import and distribution: Company C [15]

Company C, located in the Kafuri district of Khartoum North, was one of the largest importers of pharmaceutical products in Sudan, employing about 120 people. During the war, the company lost most of its existing infrastructure and inventory. Its offices and warehouses were either looted or destroyed. The Company’s location made it particularly vulnerable to the RSF armed group, which controlled much of Khartoum North in the last year. Company C’s inventory of pharmaceutical products was valued at US$15.5 million before the war began in April 2023. As of May 2024, Company C estimates that it holds less than US$3 million worth of stock. Two-thirds of the medicine stored by Company C has been stolen, and, of the rest, a significant portion is now unusable, having expired.

To continue operations, Company C relocated to the city of Wad Medani, in Gezira State, about 2 h southeast of Khartoum. The company has only been able to retain about a dozen employees, 10% of its existing workforce, and has been unable to pay redundancy dues to most of its employees beyond an initial payment. Company C estimates that its current sales capacity is about 25%. In maintaining its operations, the company has incurred additional costs for office rent, storage facility hire, staff costs, and insurance, increasing costs by the equivalent of 16 billion Sudanese pounds a month (about US$10 000 a month at April 2024 exchange rates; annual inflation is approximately 300%).

Most significantly, as a business relying on credit facilities, approximately US$8 million of outstanding payments to the Company have not been made by the pharmacies and intermediaries who had taken on stock. Due to the conflict, payments have either not been remitted or will not be remitted due to the closure or looting of the pharmacies involved. Given the subsequent devaluation of the Sudanese pound over the course of the conflict, even if outstanding obligations are reimbursed, Company C will lose a significant amount due to the exchange rate fluctuations.

About 75% of the company’s activities involved distribution of pharmaceutical goods. Company C is now unable to supply Khartoum, as well as the two states of Kordofan and the five states of Darfur. There are difficulties in supplying White Nile, Blue Nile, and Sennar States. Company C is more able to distribute products to the states of Kassala, Gedaref, River Nile, and Northern, as well as to the city of Port Sudan. This decreased distribution area is exacerbated by the unwillingness of transport companies to carry pharmaceutical products, given their reservations about their ability to adequately transport and store such goods. Moreover, Company C reports that the government-aligned forces have not helped facilitate their distribution activities, making it logistically even more complex to operate.

All these factors have led to higher costs. Company C has passed these increased costs on to consumers, even though certain drugs have government regulated price caps. As a result, Company C’s experience is that many of its products– including medicines produced in Europe that were already relatively expensive in the Sudanese market before the conflict– have become increasingly unaffordable for most patients. While precisely quantifying drug price increases is impossible given the sparsity of data, analogous price data indicates a clear trend: basic commodity food prices have increased more than four times pre-conflict levels [16].

## Discussion

This study sheds light on the effects of war on a less prominent element of the healthcare system in Sudan: the pharmaceutical manufacture and supply industry, which has produced or provided many important drugs for use in Sudan. Findings from the three case studies illustrate the dramatic and considerable impact of the war on producers and suppliers of critical drugs, which appear to have similarly affected other producers. The implication of these findings is that even if respect for health care professionals and facilities were to improve during the ongoing conflict in Sudan, or, even if the war is to end soon, the overall capacity of the Sudanese pharmaceutical industry to meet domestic need has been substantially weakened, given both damage to infrastructure and distribution systems, as well as because of the effects on the staff involved in pharmaceutical production.

While imports of drugs have long played an important role in Sudan, and continue to do so, the overall effect is that the accessibility and affordability of medicines has become sharply less so [17]. Relatively low-cost domestic manufacturing– particularly of generic drugs in common use– is not easily replaced by imports.

The war in Sudan is also, implicitly, an attack on the pharmaceutical supply chain, and the most vulnerable people within it: patients who require drugs for both acute and chronic conditions. While humanitarian assistance may alleviate some urgent needs for essential medicines in the short-term, many patients will see worse outcomes because of drug inaccessibility. Patients with easily treatable conditions may instead worsen and/or experience complications, leading to an increased burden on an already strained health system. Without medication, certain aspects of healthcare are impossible, not just difficult.

This study also highlights a gap in the literature on armed conflict and health. While we do not claim that drug manufacturing is any more important than other elements of the health system, the impact of conflict on these commercial enterprises has not been widely recognized. A more holistic approach to understanding the multiple impacts of armed conflict on health care would incorporate consideration of pharmacological production, distribution, and prescription as important dimensions of any national health system. Pharmaceutical care is particularly important in the context of a country like Sudan, which is without sufficient health care workers with advanced training. In this respect, pharmacists and pharmacies have long played a role in bridging the gap in health care provision, providing counsel and comfort rather than only dispensing medications [17–19]. The loss of pharmacists’ livelihoods further weakens the health system. Dealing with routine ailments through appropriate use of medication locally available is a typical coping mechanism of resource-poor countries; in Sudan, even this approach has been disrupted because of the inability of drug manufacturers and suppliers to continue their operations unimpeded.

### Limitations

This study is not a comprehensive or statistically representative survey of the status of the pharmaceutical industry in Sudan. While additional respondents were contacted, not all agreed to be interviewed, variously citing political and commercial sensitivities, and in some cases, difficult personal circumstances as preventing them from participating. While this is not a representative study, we sought to engage respondents who were not associated with each other, to ensure as great a spread of responses as possible. That findings are largely similar between case studies suggests the overall discussion and conclusions of the study are supported by the evidence.

Due to the ongoing conflict, the situation in Sudan is evolving quickly. While most focus is on how developments relate to wider politics and conflict, these do affect the overall operating environment for businesses such as pharmaceutical manufacturers and traders, and as the case studies suggest, how they find ways to adapt. For instance, as the conflict ebbs and flows, access / restrictions to certain states of the country are in flux, and may well deteriorate and/or improve, depending on local circumstances.

The study did not address in depth the retail pharmacy dimension of the pharmaceutical supply chain. This is because pharmacies in Sudan are both much more numerous and decentralized than manufacturers, harder to contact from outside the country given the ongoing conflict, and would be more difficult to pseudonymize while reporting important local circumstances. While the Professional Pharmacists Association has reported some data on the overall status of pharmacies in Sudan over the course of the conflict [6], this data is also incomplete.

## Conclusion

This study is the first to provide detailed accounts of the experiences of pharmaceutical manufacture and supply companies in Sudan since the onset of the war in April 2023, and is among the first to consider attacks on the pharmaceutical supply chain as part of the effects of armed conflict on health care provision. Sudan’s pharmaceutical industry, although always accounting for only a proportion of the country’s overall drug needs, has effectively stopped producing essential medicines, including antibiotics. Moreover, existing distribution systems for drug companies have been severely compromised, leading to drug unavailability and/or higher costs for pharmacies and patients. While some companies have been able to adapt and find ways to continue operations, respondents report that this comes at high cost and with severe limitations. Given the costs and the specialized nature of the industry, setbacks in the pharmaceutical industry are not easily remedied. This disruption of the pharmaceutical supply chain is yet another blow to the already precarious health care system in Sudan.

## Data Availability

Summarized interview data analyzed in this study is available from the corresponding author upon reasonable request.

## Abbreviations

NMPB: National Medicines and Poison Board
NMSF: National Medical Supplies Fund
NSAIDs: Nonsteroidal anti-inflammatory drugs
RSF: Rapid Support Forces
